# 

**Published:** 1802-03

**Authors:** 


### To CORRESPONDENTS.

*Communications are received from Doctors Patterson, Bellamy, Winterbottom, Woodforde, Noehden, Girdlestone; Messrs Crowfoot, Blegborough, Hill, Reece, Mills, Ferguson, White, Gaisford, Coley, Robinson, Tatam, Obstetricus, Honestus, A. R. and S. G. together with an Account of several Country Hospitals.*

